# Targeting lysyl oxidase like 2 attenuates OVA-induced airway remodeling partly via the AKT signaling pathway

**DOI:** 10.1186/s12931-024-02811-4

**Published:** 2024-06-01

**Authors:** Rong Zeng, Dong Zhang, Jintao Zhang, Yun Pan, Xiaofei Liu, Qian Qi, Jiawei Xu, Changjuan Xu, Shuochuan Shi, Junfei Wang, Tian Liu, Liang Dong

**Affiliations:** 1 Department of Respiratory, Shandong Provincial Qianfoshan Hospital, Shandong University, Jinan, China; 2 Department of Respiratory, The First Affiliated Hospital of Shandong First Medical University, Shandong Institute of Respiratory Diseases, Jinan, China; 3 Department of Respiratory, Shandong Qianfoshan Hospital, School of Clinical and Basic Medical Sciences, Shandong First Medical University & Shandong Academy of Medical Sciences, Jinan, China; 4 Department of Respiratory and Critical Care Medicine, Qilu hospital of Shandong University, Jinan, China

**Keywords:** Asthma, Airway remodeling, LOXL2, Extracellular matrix, EMT

## Abstract

**Background:**

Airway epithelium is an important component of airway structure and the initiator of airway remodeling in asthma. The changes of extracellular matrix (ECM), such as collagen deposition and structural disturbance, are typical pathological features of airway remodeling. Thus, identifying key mediators that derived from airway epithelium and capable of modulating ECM may provide valuable insights for targeted therapy of asthma.

**Methods:**

The datasets from Gene Expression Omnibus database were analyzed to screen differentially expressed genes in airway epithelium of asthma. We collected bronchoscopic biopsies and serum samples from asthmatic and healthy subjects to assess lysyl oxidase like 2 (LOXL2) expression. RNA sequencing and various experiments were performed to determine the influences of LOXL2 knockdown in ovalbumin (OVA)-induced mouse models. The roles and mechanisms of LOXL2 in bronchial epithelial cells were explored using LOXL2 small interfering RNA, overexpression plasmid and AKT inhibitor.

**Results:**

Both bioinformatics analysis and further experiments revealed that LOXL2 is highly expressed in airway epithelium of asthmatics. In vivo, LOXL2 knockdown significantly inhibited OVA-induced ECM deposition and epithelial-mesenchymal transition (EMT) in mice. In vitro, the transfection experiments on 16HBE cells demonstrated that LOXL2 overexpression increases the expression of N-cadherin and fibronectin and reduces the expression of E-cadherin. Conversely, after silencing LOXL2, the expression of E-cadherin is up-regulated. In addition, the remodeling and EMT process that induced by transforming growth factor-*β*1 could be enhanced and weakened after LOXL2 overexpression and silencing in 16HBE cells. Combining the RNA sequencing of mouse lung tissues and experiments in vitro, LOXL2 was involved in the regulation of AKT signaling pathway. Moreover, the treatment with AKT inhibitor in vitro partially alleviated the consequences associated with LOXL2 overexpression.

**Conclusions:**

Taken together, the results demonstrated that epithelial LOXL2 plays a role in asthmatic airway remodeling partly via the AKT signaling pathway and highlighted the potential of LOXL2 as a therapeutic target for airway remodeling in asthma.

**Supplementary Information:**

The online version contains supplementary material available at 10.1186/s12931-024-02811-4.

## Background

Asthma is a common chronic airway disease affecting 1-29% of the population in different countries [1, 2]. It is characterized by paroxysmal symptoms of wheeze, chest tightness, shortness of breath, and cough, together with variable expiratory airflow limitation [3]. Currently, most asthmatics can be well controlled with the rational use of corticosteroids, bronchodilators, and biologics [4]. However, due to poor response to above treatments, some patients have recurrent episodes and finally develop persistent airflow limitation [4, 5]. Airway remodeling, also known as airway structural changes including extracellular matrix (ECM) accumulation, smooth muscle hyperplasia, and subepithelial fibrosis, is a major cause of persistent airflow limitation in asthmatics [3, 5]. Hence, it is necessary to identify potential targets that can delay or inhibit the development of airway remodeling.

The ECM is an intricate network composed of structural and non-structural components derived from different cells, such as epithelial cells, smooth muscle cells and fibroblasts [6, 7]. Deposition, disorganization and stiffness of ECM are important pathological features of tissue remodeling, which can affect the phenotype and function of resident cells in forms of mechanotransduction and outside-in signaling [8, 9]. Correspondingly, a growing number of researchers pay attention to mediators capable of modulating ECM, and the benefits of targeted therapies in disease models.

Lysyl oxidase like 2 (LOXL2), a member of lysyl oxidase family, plays a pivotal role in catalyzing the cross-linking of elastin and collagen, which is essential for ECM homeostasis [10]. It is reported that mechanical stimuli, inflammation, and hypoxia all can induce the upregulation of LOXL2 [11–13], and as a consequence, the pathological process of fibrosis or remodeling in various diseases, such as heart failure, cancer, pulmonary arterial hypertension, and organ fibrosis (e.g. liver, lung, and renal), are aggravated [11, 14–18]. For example, Yang et al. found that areas with profound interstitial fibrosis of heart failure patients are always accompanied by high LOXL2 expression, and the level of which is closely associated with diastolic dysfunction [11]. Subsequently, LOXL2 was testified to be significantly increased in several fibrotic animal models and promoted the activation of fibroblasts and myofibroblasts, which in turn produced more ECM [9, 16–18]. The small molecule inhibitors selectively targeting LOXL2 have shown considerable effect in inhibiting fibrosis progression [16, 18, 19]. Recently, Ramis et al. observed the level of LOXL2 in airway smooth muscle (ASM) cells and smooth muscle bundles was dramatically upregulated in asthmatics and the suppression of LOXL2 could reduce ECM stiffness and ASM thickness [20]. Notably, airway epithelium is the initiator of airway remodeling in asthma, in which the expression level of LOXL2 is also significantly elevated based on the analyses of RNA expression files from the database of Gene Expression Omnibus (GEO) (https://www.ncbi.nlm.nih.gov/geo/). Thus, further exploration of the relationship and potential mechanisms between LOXL2 and asthma may provide meaningful insights for developing promising treatments.

## Materials and methods

### Identification of differentially expressed genes (DEGs)

We identified the datasets of GSE179156 and GSE40374 from the GEO database. In GSE179156, the transcriptome sequencing of bronchial epithelium from healthy controls (*n* = 29) and asthmatics with no treatment (*n* = 38) was analyzed with GEO2R to obtain the DEGs (based on |Fold change|≥1.5 and *P* value < 0.05). Meanwhile, the same analysis was performed on M-BE cells treated with or without transforming growth factor-β1 (TGF-β1) in GSE40374. The list of 1026 ECM-related genes was downloaded from the Molecular Signatures Database (https://www.gsea-msigdb.org/gsea/msigdb), Additional file 1: Table S1. The STRING database (https://www.string-db.org/) was used to analyzed the interaction of overlapping genes. Subsequently, the result was imported to the Cytoscape software to identify the key DEGs.

### Subjects and specimen sampling

Patients with asthma diagnosed based on the Global Initiative for Asthma (updated in 2022) and healthy controls were recruited at the respiratory clinic of the local hospital. The clinical data and 5 mL fasting venous blood samples of all participants were collected. After centrifuging the procoagulant tubes containing blood samples at 3000 rpm for 15 minutes, we absorbed the supernatant and immediately stored them at -80°C. Bronchial epithelial specimens of asthma patients were obtained by bronchoscopic biopsy, while subjects with suspected intra-airway lesions but no abnormal pathological findings provided the normal biopsy specimens. The clinical characteristics of subjects that donated the bronchial epithelial specimens are shown in Additional file 1: Table S2. All subjects in this study were informed of the study’s purpose and signed the written consent.

### Enzyme-linked immunosorbent assay (ELISA)

The commercial ELISA kit (EK1391; Boster, Wuhan, China) was used to measure the level of LOXL2 in serum. The experiment procedure was carefully carried out according to the manufacturer’s instruction.

### Cell culture and treatment

Human bronchial epithelial (16HBE) cells were purchased from Fuheng Biotechnology (Shanghai, China) and cultured in Keratinocyte Medium (KM; ScienCell, Carlsbad, CA, USA) supplemented with 1% Keratinocyte Growth Supplement (KGS; ScienCell). The cells were incubated at 37°C with a carbon dioxide content of 5%. The human TGF-β1 protein (PRP100190, Abbkine, Wuhan, China) was used to stimulate 16HBE cells at different concentrations and time points. To investigate the function and mechanism of LOXL2, we transfected 16HBE cells with control vector or overexpression LOXL2 vector (GenePharma, Shanghai, China), small interfering RNA (siRNA) negative control or LOXL2 siRNA (GenePharma, shanghai, China) using EndoFectin^TM^-Max (GeneCopoeia) according to the manufacturer’s instruction. The LOXL2 siRNAs sequences are as follows:

siRNA-1:5’-GGGCAGAAGAGGAAGCACATTUGUGCUUCCUCUUCUGCCCTT-3’;

siRNA-2:5’-AGAUAGAGAACCUGAAUAUTTAUAUUCAGGUUCUCUAUCUTT-3’;

siRNA-3:5’-AGGUGCUGGUGGAGAGAAATTUUUCUCUCCACCAGCACCUTT-3’.

After the experiments of plasmid transfection, the 16HBE cells also were treated with AKT inhibitor (MK2206, Selleck, USA) to explore the potential mechanism of LOXL2.

### Animal model construction and protocols

Female C57BL/6 mice weighing about 20 g at 7-8 weeks were purchased from PengYue Laboratory Animal Breeding Co., Ltd. (Jinan, China). To establish the chronic asthma models, mice were treated in two stages. First, they were sensitized by intraperitoneal injection of ovalbumin (OVA) solution (20ug in 200 μL phosphate-buffered saline; Sigma-Aldrich) mixed with Al(OH)_3_ (2mg) at days 0, 7, and 14. Then, the mice were challenged by inhaling 3% OVA aerosol on alternate days from 21 to 58 days. The lentivirus (LV)-packed LOXL2 short hairpin RNA (shRNA) or negative control shRNA (LV-shNC) were injected into mice via tail vein. Based on our team’s previous study, the lentivirus was injected into mice in three separate doses between days 21 and 58. Correspondingly, we divided the mice into 4 groups (6 mice in each group): 1) LV2-shNC group; 2) LV2-shLOXL2 group; 3) OVA+LV2-shNC group; 4) OVA+LV2-shLOXL2 group. Referring to our team’s previous study, mouse airway resistance under the stimulation of methacholine was measured using flexiVent (Scireq).

### RNA-sequencing and results analysis

The left lung tissues of OVA+LV2-shNC and OVA+LV2-shLOXL2 groups were separated and immediately sent to BGI Genomics (Shenzhen, China) for RNA sequencing. The DEGs were analyzed and downloaded from the online platform of BGI Genomics (https://biosys.bgi.com). Subsequently, we used DAVID database (https://david.ncifcrf.gov/home.jsp) to implement the enrichment analyses of Gene Ontology (GO) and Kyoto Encyclopedia of Genes and Genomes (KEGG). The filtering criterion with statistical significance is *P* value < 0.05.

### Quantitative real-time-polymerase chain reaction (qRT-PCR)

We extracted the total RNA from 16HBE cells with the RNA Fast 200 RNA Extraction kit (Fastagen Biotech, Shanghai, China). Based on the purity and concentration of RNA was measured by the spectrophotometer, we determined the volume for reverse transcription of each sample and then synthesized cDNA using reverse transcription reagent (Vazyme, Nanjing, China). The level of mRNA was measured with a Bio-Rad CFX96 Real-Time system using the ChamQ Universal SYBR qPCR Master Mix (Vazyme, Nanjing, China). Additional file 1: Table S3 provided primer sequences in detail.

### Western blotting

After the incubation in the radioimmunoprecipitation assay buffer (Solarbio, Beijing, China) containing phenylmethanesulfonyl fluoride (Solarbio, Beijing, China), the total protein of lung tissues or cells was extracted for subsequent quantification and electrophoresis. Briefly, the total protein was segregated on 10% sodium dodecyl sulfate polyacrylamide gel electrophoresis gels, and transferred to polyvinylidene difluoride membranes. The membranes were blocked at room temperature for 1.5 hours using 5% bovine serum albumin, and then incubated with different primary antibodies overnight at 4°C. The primary antibodies used in this study are as follows: anti-LOXL2 (1:1,000; Abcam, Cambridge, MA, USA), anti-GAPDH (1:5,000; HUABIO, Hangzhou, China), anti-E-cadherin (1:1,000; Cell Signaling Technology, Danvers, MA, USA), anti-N-cadherin (1:1,000; Cell Signaling Technology), anti-fibronectin (1:1,000; HUABIO), anti-collagen I (1:1,000; Abcam), anti-alpha smooth muscle actin (α-SMA; 1:1,000; HUABIO), anti-AKT (1:1,000; HUABIO), anti-p-AKT (1:1,000; Cell Signaling Technology). After washing with tris-buffered saline containing 0.1% Tween 20 (TBST), we incubated the membranes with goat anti-rabbit IgG (H+L) HRP (1:5,000; HUABIO) for 1.5 hours at room temperature. Then, the membranes were washed again with TBST, and finally visualized with enhanced chemiluminescent reagent (Vazyme, Nanjing, China).

### Migration assay

The 16HBE cells in 6-well plates that have been treated under different experimental conditions, were digested with 0.25% Trypsin-EDTA and re-suspended in KM without KGS. KM suspension (150 μL) comprising about 1 × 10^6^ cells was inoculated into 8 μm transwell chambers, and 600 μL of KM  supplemented with 1% KGS was added to the wells under the chambers. The cells were cultivated in the chamber for 24 hours. After fixing with 4% paraformaldehyde and staining with 0.5% crystal violet, we used cotton swabs to wiped the non-migrated cells in the upper chambers. Five visual fields (100×) were randomly selected to observe the cells penetrated the membrane. The software of Image J was used to count the number of migrated cells.

### Histopathological evaluation and Immunohistochemistry (IHC) staining

The lung tissues were separated and immediately fixed in 4% paraformaldehyde for 24 hours. Subsequently, they were embedded in paraffin and cut into 5-μm-thick paraffin sections for different staining. For example, Masson’s staining was implemented to assess collagen deposition around airways, IHC staining was used to observe the expression of remodeling and EMT markers. Specifically, after the deparaffinization and dehydration, sections of lung tissue were repaired with citrate unmasking solution and treated with 3% H_2_O_2_ to block the activity of endogenous peroxidase. To avoid non-specific binding, the sections were blocked with goat serum for 1 hour. Then, the primary antibodies including anti-LOXL2 (1:200; Abcam), anti-E-cadherin (1:200; Cell Signaling Technology), and anti-α-SMA (1:200; HUABIO) were used to incubate the samples overnight at 4°C. The next day, biotinylated anti-rabbit IgG antibody was used to react with the primary antibody. Finally, the color depth was observed at a certain time after the sections were exposed to substrate chromogen mixture (BOSTER, Wuhan, China).

### Immunofluorescence (IF) staining

The experimental procedure for IF staining was similar to that for IHC staining until the sections were incubated with primary antibody. Primary antibodies used for IF staining are as follows: anti-LOXL2 (1:200; Abcam), anti-N-cadherin (1:200; Cell Signaling Technology) and anti-collagen I(1:200; Abcam). Following day, the sections were incubated with secondary antibody (1:200; Abbkine Scientific Co., Ltd.) for 1 hour at room temperature and then the nuclei were stained using 4’,6-diamidino-2-phenylindole (1:200) for 8 minutes.

### Statistical analysis

The data conforming to normal distribution was presented as mean±SD, otherwise it was described as medians with interquartile ranges. Statistical analysis between two groups were implemented via Student’s *t* test (normal distributed parameters) or Mann-Whitney test (non-normal distributed parameters). The significant analysis for multiple groups of data meeting normal distribution was implemented using one-way analysis of variance (ANOVA) followed by Dunnett’s test or Tukey’s post hoc test. Spearman’s correlation coefficient was used to evaluate the correlation between serum LOXL2 and pulmonary function indexes. The analyses of the above data were completed by GraphPad software (version 8.0) and SPSS software (version 25.0). A two tailed *P* value of < 0.05 was considered statistically significant.

## Results

### Identification of DEGs

To identify underlying targets associated with airway remodeling in asthma, we analyzed the two microarray datasets of GSE179156 and GSE40374 from GEO database. Based on the standards of |Fold change|≥1.5 and *P* value < 0.05, 6473 DEGs in GSE40374 and 1582 DEGs in GSE179156 were screened (Additional file 1: Fig. S1A-B). It is reported that more and more ECM components play critical roles as modulators of inflammatory and remodeling events. For example, some proteoglycans can interact with various partners (growth factors, morphogens, chemokines, proteases, etc.) to affect multiple cellular functions and promote the persistence of lung inflammation and fibrosis [21, 22]. Therefore, we intersected 1026 ECM-related genes with DEGs in the above two datasets and identified 44 ECM-related DEGs (Additional file 1: Fig. S1C). Next, the 44 overlapping genes were inputted into the database of STRING for in-depth analysis, and the result showed there were intricate relationships between them (Additional file 1: Fig. S1D). Meanwhile, based on the interaction network that analysed by STRNG database, the five algorithms (MCC, MNC, EPC, DMNC and Degree) in Cytoscape software were adopted to find key genes. Remarkably, LOXL2 was the only gene that ranked in the top 10 of each algorithms and highly expressed in normal lung tissue and airway epithelium (Additional file 1: Fig. S1E-G). As shown in Additional file 1: Fig. S1H-I, LOXL2 was up-regulated in both bronchial epithelium of asthmatics and M-BE cells treated with TGF-β1.

### LOXL2 is increased in airway epithelium of patients with asthma and chronic asthma mouse model

**Fig. 1:**
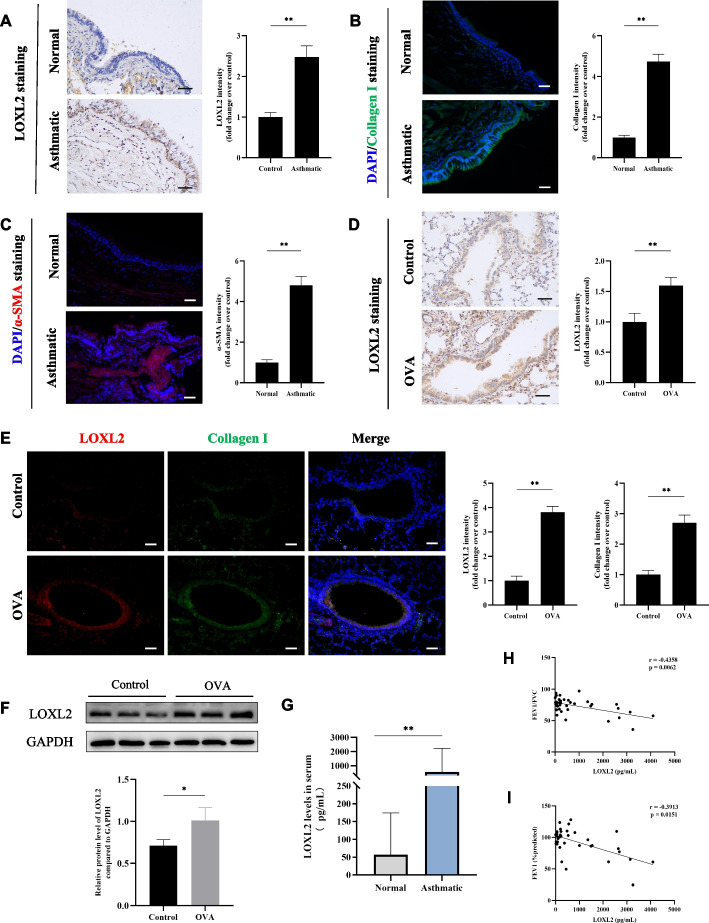
Up-regulated expression of LOXL2 in asthmatics and chronic asthma mice. **A** Expression and quantification of LOXL2 by immunohistochemistry in the airway epithelium of asthma patients and healthy controls. **B**, **C** The levels and quantifications of collagen I (**B**, green) and α-SMA (**C**, red) were measured by immunofluorescence around the airway of asthma patients and healthy controls. **D** Immunohistochemistry detection of LOXL2 in chronic asthma mice. **E** Coimmunostaining of LOXL2 (red) and collagen I (green) in chronic asthma mice. **F** Western blotting detection of LOXL2 in chronic asthma mice. **G** Serum levels of LOXL2 in healthy controls and asthmatics. **H** Correlation analysis between serum LOXL2 and FEV1/FVC (*r* = -0.4358, *P* = 0.0062). **I** Correlation analysis between serum LOXL2 and FEV1 (%predicted) values  (*r* = -0.3913, *P* = 0.0151). Scale bar, 50 μm. Student’s t test, Mann-Whitney test and Spearman correlation analysis were used. Data are shown as the means ± SD of three independent experiments. ^*^P < 0.05, ^**^P < 0.01 versus the control group

Based on the results of bioinformatics analysis, IHC staining was performed on bronchial epithelial biopsy specimens of asthma patients and healthy controls, and then demonstrated LOXL2 was highly expressed in airway epithelium of asthmatics (Fig. 1A). Meanwhile, the results of IF staining also demonstrated that there were increased remodeling markers (Collagen I and α-SMA) around the airway (Fig. 1B-C). We further confirm the expression level and location of LOXL2 in the OVA-induced chronic asthma models. Specifically, western blotting showed that LOXL2 was up-regulated in the lung tissues of OVA-treated mice (Fig. 1F), and IHC staining showed that LOXL2 was significantly increased in the airway epithelium of OVA-treated mice (Fig. 1D). Using the coimmunostaining of LOXL2 and collagen I, we found that they were co-localized in the airway epithelium and significantly elevated in OVA-treated mice (Fig. 1E).

Given that LOXL2 can be detected in peripheral blood, serum samples from 27 asthmatics and 11 healthy controls were collected for ELISA. Additional file 1: Table S4 demonstrated the baseline characteristics of all participants. As shown in Fig. 1G, asthmatics had higher serum levels of LOXL2 compared with healthy controls. The correlation analyses of spearman indicated that the serum levels of LOXL2 were negatively correlated with forced expiratory volume in one second (FEV1) to forced vital capacity ratio (FVC) (*r* = -0.4358, *P* = 0.0062) and FEV1%predicted (*r* = -0.3913, *P* = 0.0151) (Fig. 1H-I).

### The expression of LOXL2 is up-regulated in bronchial epithelial cells with TGF-β1 treatment

**Fig. 2:**
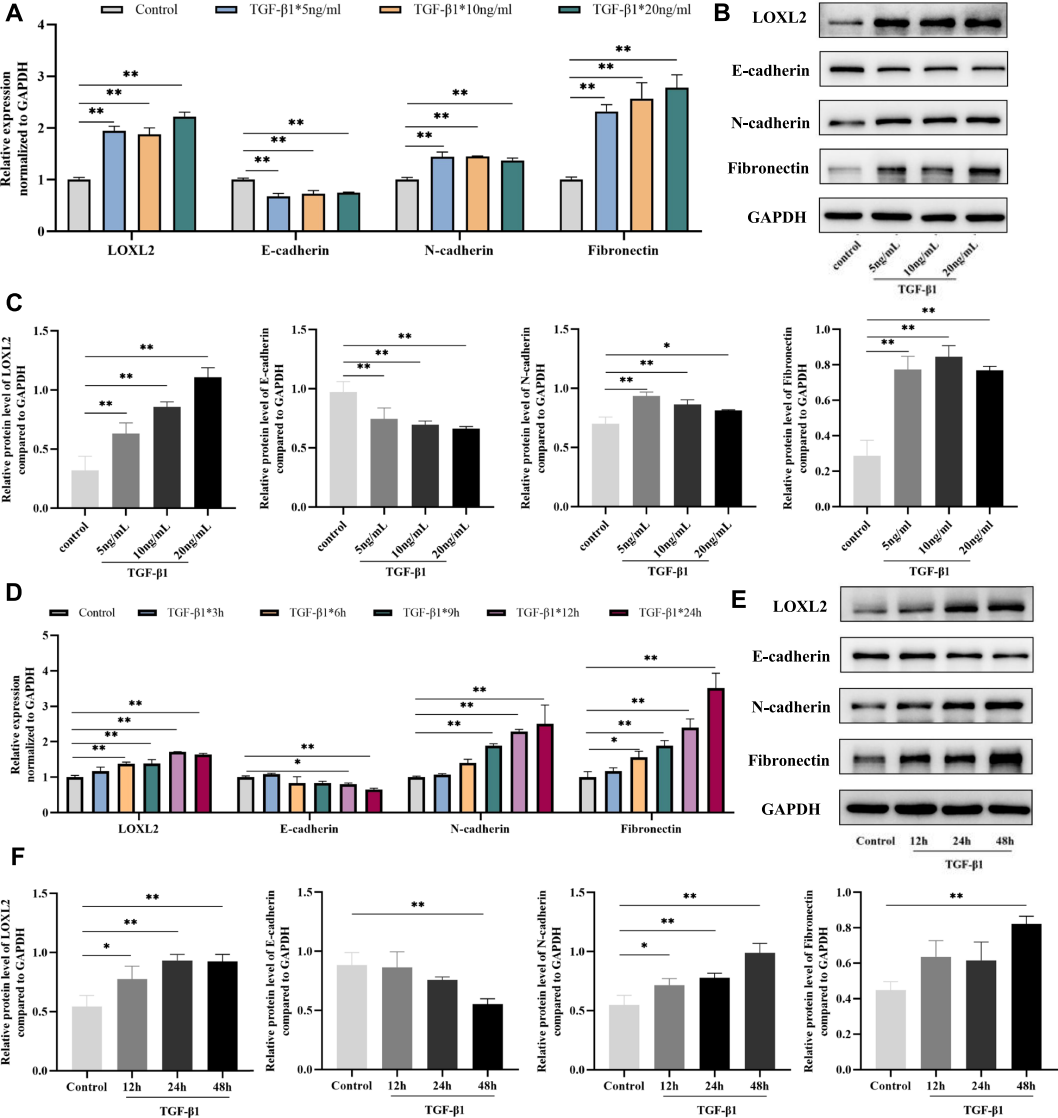
LOXL2 is significantly increased with TGF-*β*1 treatment in bronchial epithelial cells. **A** The mRNA levels of LOXL2, E-cadherin, N-cadherin, and fibronectin after treatment with different concentrations of TGF-*β*1 for 24 hours. **B**, **C** The protein levels and quantifications of LOXL2, E-cadherin, N-cadherin, and fibronectin after treatment with different concentrations of TGF-*β*1 for 48 hours. **D** The mRNA levels of LOXL2, E-cadherin, N-cadherin, and fibronectin after treatment with 5 ng/mL TGF-*β*1 at different time points (0, 3, 6, 9, 12, and 24 hours). **E**, **F** The protein levels and quantifications of LOXL2, E-cadherin, N-cadherin, and fibronectin after treatment with 5 ng/mL TGF-*β*1 at different time points (0, 12, 24, and 48 hours). One-way analysis of variance followed by Dunnett’s test was used. Data are shown as the means ± SD of three independent experiments. ^*^P < 0.05, ^**^P < 0.01 versus the control group

Extensive prior studies have reported that TGF-*β*1 is a pivotal driver of EMT and airway remodeling in asthma. The bioinformatics analysis of GSE40374, in which M-BE cells were treated with or without TGF-β1, suggested that LOXL2 is up-regulated in response to the stimulation of TGF-β1. We further confirmed that the mRNA and protein levels of LOXL2 were significantly increased in 16HBE cells under different concentrations (0, 5, 10, 20 ng/mL) and time points (0, 3, 6, 9, 12 and 24 hours in mRNA; 0, 12, 24, 48 hours in protein) of TGF-β1 stimulation (Fig. 2A-F). As found in previous studies, after stimulating with TGF-β1, the protein and mRNA levels of E-cadherin significantly decreased in 16HBE cells, while the protein and mRNA levels of N-cadherin and fibronectin significantly increased (Fig. 2A-F).

### LOXL2 knockdown attenuates ECM deposition during OVA-induced airway remodeling

**Fig. 3:**
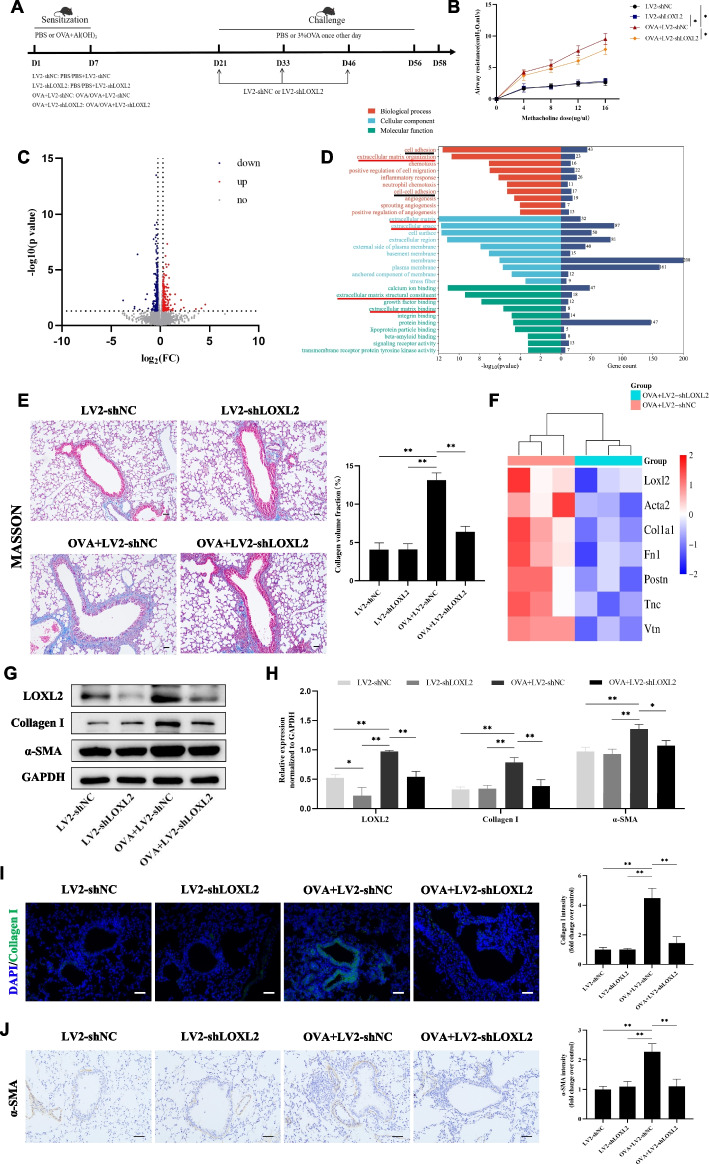
Knockdown LOXL2 mitigates ECM deposition in the OVA-induced chronic asthma models. **A** The flowchart and groups of animal experiments. **B** Assessment of mouse airway resistance in each group using different doses of methacholine. **C** Volcano plot of DEGs (|log_2_FC|≥0.262, *P* < 0.05) between OVA+LV2-shNC and OVA+LV2-shLOXL2 groups. Red represents upregulated DEGs, and blue represents downregulated DEGs. **D** Barplot of GO enrichment analysis (Top 10 enriched terms with *P* value <0.05). **E** Collagen deposition around the airway epithelium of mouse models was evaluated with Masson staining. Scale bar, 50 μm. **F** Cluster heatmap of several ECM-related genes based on the RNA-seq data (*P* value<0.05). **G**-**H** The expression levels and quantitative analyses of ECM proteins (collagen I and α-SMA) in mouse lung tissues after different treatment. **I** Collagen I around the airway epithelium of mouse models was observed by Immunofluorescence staining. Scale bar, 50 μm. **J** The expression of α-SMA around the airway was evaluated by Immunohistochemical staining. Scale bar, 50 μm. One-way analysis of variance followed by Tukey’s post hoc test. Data are shown as the means ± SD of three independent experiments. ^*^P < 0.05, ^**^P < 0.01 versus the corresponding group

In order to explore the potential functions of LOXL2 in chronic asthma models, lentiviral shRNA vectors targeting LOXL2 were constructed and injected into mice through tail vein. Figure 3A presented the flow of chronic asthma model construction and time points of lentivirus intervention. We extracted mouse lung tissue protein and performed western blotting to verify the effectiveness of interference (Additional file 1: Fig. S2A). The IF staining of lung tissue sections also showed that LOXL2 was elevated in airway epithelium of OVA-induced asthma models. After lentivirus treatment, the OVA-induced LOXL2 expression in airway epithelium was indeed inhibited (Additional file 1: Fig. S2B-C). The assessment of airway resistance showed that there were marked airway hyperresponsiveness in OVA-treated mice, which could be partially alleviated in the condition of LOXL2 knockdown (Fig. 3B).

Furthermore, RNA-sequencing of OVA+LV2-shNC and OVA+LV2-shLOXL2 groups were performed to observe transcriptome changes in the lung of chronic asthma models. According to the filter criteria (|log_2_FC|≥0.262 and *P* value < 0.05), we identified 438 DEGs between the two groups. Results were visualized with volcano plot and included 188 up-regulated and 250 down-regulated genes (Fig. 3C). Subsequently, GO analysis of DEGs indicated that enrichment items mainly focused on ECM organization, extracellular space, ECM structural constituent, and ECM binding (Fig. 3D). The cluster heatmap of selected DEGs also provided further evidence that various ECM genes (COL1A1, FN1, ACTA2, and POSTN) were significantly down-regulated in OVA+LV2-shLOXL2 group (Fig. 3F). Concomitantly, Masson staining suggested that collagen deposition around airway epithelium was increased in OVA-induced chronic asthma mice and then markedly decreased after treatment with LV2-shLOXL2 (Fig. 3E). In terms of ECM proteins in lung tissues, western blotting showed that LOXL2 knockdown could partially reduce OVA-induced up-regulation of Collagen I and α-SMA (Fig. 3G-H). In addition, the results of IF or IHC staining for above markers were consistent with those of western blotting (Fig. 3I-J).

### LOXL2 knockdown also inhibits EMT during OVA-induced airway remodeling

**Fig. 4:**
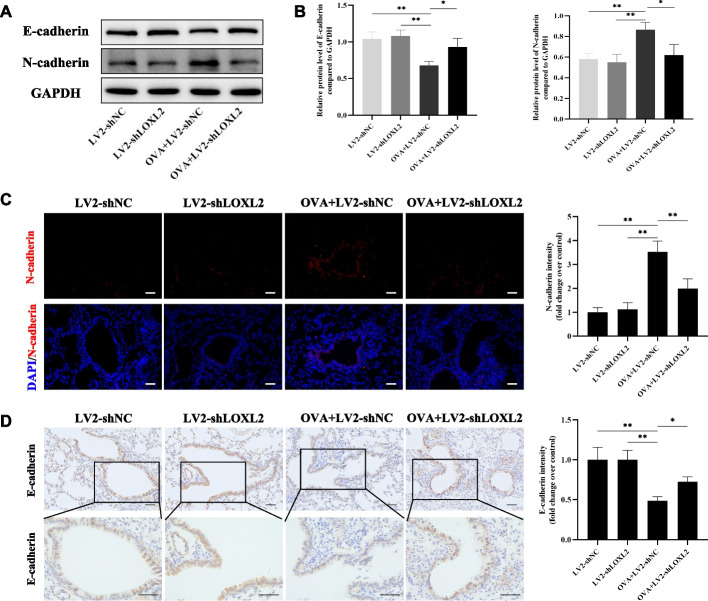
Knockdown LOXL2 inhibits the EMT in OVA-induced chronic asthma models. **A**, **B** The expression levels of EMT indicators (E-cadherin and N-cadherin) in mouse lung tissues after different treatment, and subsequent quantitative analyses. **C** The Immunofluorescence staining was used to observe the expression of N-cadherin around the mouse airway epithelium. Scale bar, 50 μm. **D** The Immunohistochemical staining was used to evaluate the expression of E-cadherin around the mouse airway epithelium. Scale bar, 50 μm. One-way analysis of variance followed by Tukey’s post hoc test. Data are shown as the means ± SD of three independent experiments. ^*^P < 0.05, ^**^P < 0.01 versus the corresponding group

Remarkably, biological process of cell-cell adhesion, one of the early and core changes during EMT, was also significantly affected after LOXL2 knockdown in chronic asthma models (Fig. 3D). Thus, we performed western blotting, IF, and IHC staining to determine the alterations of epithelial indicator (E-cadherin) and mesenchymal indicator (N-cadherin). In terms of protein levels in lung tissues, LOXL2 knockdown could significantly reversed OVA-induced down-regulation of E-cadherin and up-regulation of N-cadherin (Fig. 4A-B). In addition, results of IF or IHC staining for the two markers in airway epithelium were same as those of western blotting (Fig. 4C-D). Overall, it is reasonable to infer that targeting LOXL2 can effectively alleviate OVA-induced ECM deposition and EMT.

### LOXL2 plays an important role in the EMT and remodeling of bronchial epithelial cells

**Fig. 5:**
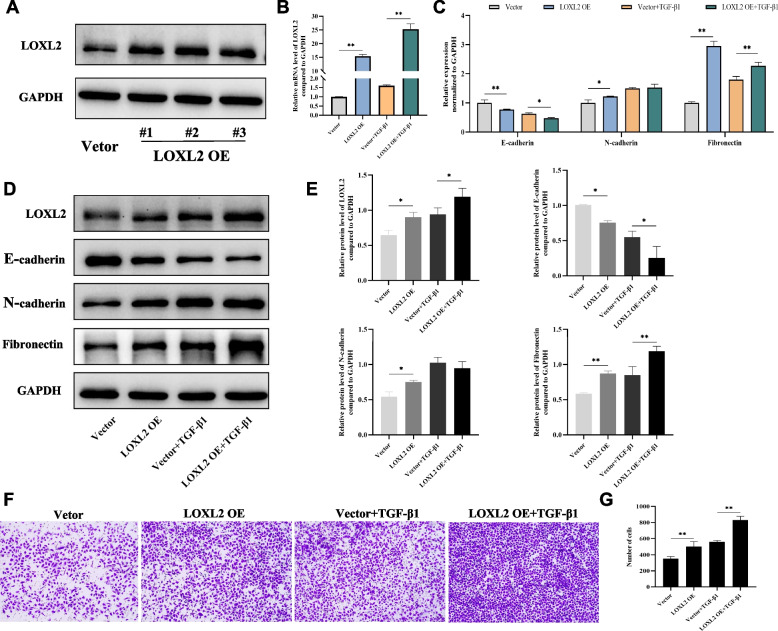
LOXL2 overexpression in bronchial epithelial cells promotes the process of EMT, remodeling and migration. **A** Protein level of LOXL2 is significantly increased after LOXL2 plasmid transfection. **B**, **C** mRNA levels of LOXL2, E-cadherin, N-cadherin, and fibronectin after LOXL2 plasmid transfection and TGF-*β*1 treatment. **D**, **E** Protein expression and quantitative analyses of LOXL2, E-cadherin, N-cadherin, and fibronectin after LOXL2 plasmid transfection and TGF-*β*1 treatment. **F**, **G** The effects of LOXL2 overexpression alone and together with TGF-*β*1 treatment on migration ability of 16HBE cells. Data are shown as the means±SD of three independent experiments. ^*^P < 0.05, ^**^P < 0.01 versus the corresponding group

Considering the variation tendency of indicators (LOXL2, E-cadherin, N-cadherin and fibronectin) in above experiments, we selected 5 ng/mL of TGF-β1 to stimulate 16HBE cells for 24 hours and 48 hours, and then evaluated those mRNA and protein levels, respectively. Firstly, we constructed the plasmid vector to overexpress LOXL2 in 16HBE cells. As expected, both western blotting and qRT-PCR suggested that transfection of LOXL2 plasmid was successful (Fig. 5A-B). To explore the effects of LOXL2 alone and together with TGF-β1 on EMT and airway remodeling, the experiments were divided into four groups: 1) vector group; 2) LOXL2 overexpression (LOXL2 OE) group; 3) vector+TGF-β1 group; 4) LOXL2 OE+TGF-β1 group. Results showed that overexpression of LOXL2 alone appeared to promote the emergence of EMT and remodeling in 16HBE cells, concretely speaking, the expression level of E-cadherin was decreased, while the expression levels of N-cadherin and fibronectin were increased (Fig. 5C-E). Notably, LOXL2 overexpression also amplified TGF-β1-induced down-regulation of E-cadherin and up-regulation of fibronectin (Fig. 5C-E). Besides, we observed that LOXL2 overexpression increased the number of cells that successfully migrated compared with the vector group. Similarly, more cells migrated to lower chamber in group of LOXL2 OE+TGF-β1 than in group of vector+TGF-β1 (Fig. 5F-G).

**Fig. 6:**
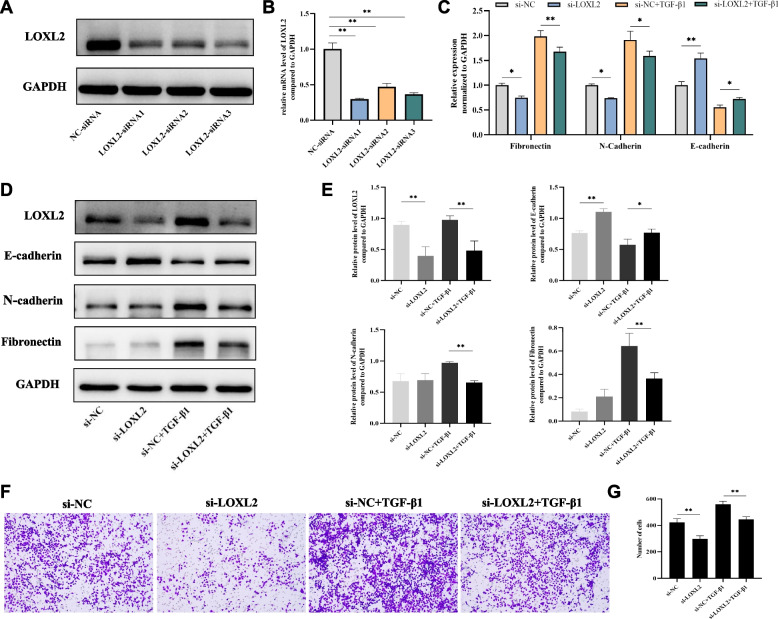
Effects of LOXL2 silencing on EMT, remodeling indicators and migration ability in bronchial epithelial cells. **A**, **B** Both protein and mRNA expression of LOXL2 are inhibited by siRNA transfection. **C** mRNA levels of E-cadherin, N-cadherin, and fibronectin after LOXL2 silencing and TGF-*β*1 treatment. **D**, **E** Protein expression and quantitative analyses of E-cadherin, N-cadherin, and fibronectin after LOXL2 silencing and TGF-*β*1 treatment. **F**, **G** The effects of LOXL2 silencing alone and together with TGF-*β*1 treatment on migration ability of 16HBE cells. Data are shown as the means ± SD of three independent experiments. ^*^P < 0.05, ^**^P < 0.01 versus the corresponding group

Next, to further demonstrate the role of LOXL2 on EMT and remodeling of bronchial epithelial cells, three siRNAs (LOXL2-siRNA1, LOXL2-siRNA2 and LOXL2-siRNA3) targeting LOXL2 were synthesized and transfected into 16HBE cells. Given the transfection efficiency evaluated by western blotting and qRT-PCR, the LOXL2-siRNA3 was selected for the subsequent experiments (Fig. 6A-B). We observed that after silencing LOXL2 in 16HBE cells, the expression level of E-cadherin was up-regulated, while the expression levels of N-cadherin and fibronectin was decreased (Fig. 6C-E). Not only that, but LOXL2 knockdown notably decreased the levels of TGF-β1-induced N-cadherin and fibronectin expressions in 16HBE cells (Fig. 6C-E). Meanwhile, the expression level of E-cadherin in si-LOXL2+TGF-β1 group was higher than that in si-NC+TGF-β1 group (Fig. 6C-E). In contrast to the results of LOXL2 overexpression experiment, LOXL2 silencing significantly inhibited the migration ability of 16HBE cells, that is, the number of cells that successfully migrated was significantly less than that of si-NC group (Fig. 6F-G). Additionally, compared to si-NC+TGF-β1 group, the migrated ability of 16HBE cells in the group of si-LOXL2+TGF-β1 was also remarkably weakened (Fig. 6F-G). From the above results, it can be seen that LOXL2 is critical for the process of EMT and remodeling in airway epithelium.

### The roles of LOXL2 in airway epithelium are mediated partly via the AKT signaling pathway

**Fig. 7:**
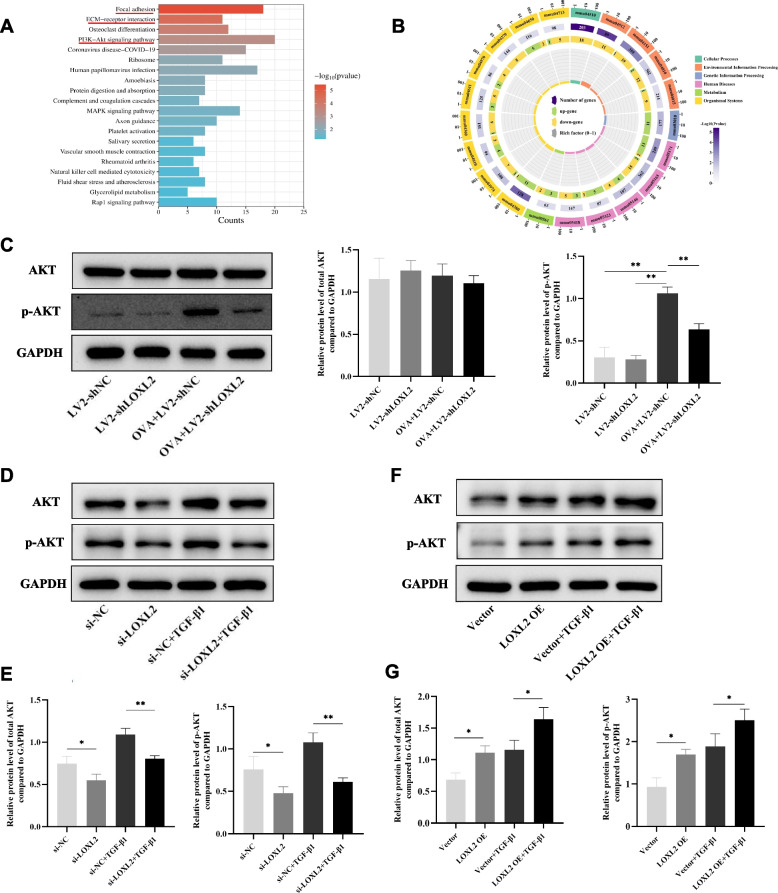
LOXL2 modulates the AKT signaling pathway. **A** KEGG pathway analysis of DEGs based on the RNA-sequencing data. Top 20 enriched terms with *P* value < 0.05 are demonstrated in the barplot. **B** Circular map of KEGG pathway enrichment. The enriched items are shown in the first circle outside. Color depth in the second circle represents the significance of enriched pathways. The number of enriched DEGs are presented in the third circle. **C** Representative western blot images of AKT and p-AKT levels in mouse lung tissues from different groups. **D** Representative western blot images of AKT and p-AKT levels after LOXL2 silencing and TGF-*β*1 treatment in 16HBE cells. **E** Relative protein levels of AKT and p-AKT compare to GAPDH after LOXL2 silencing and TGF-*β*1 treatment in 16HBE cells. **F** Representative western blot images of AKT and p-AKT levels after LOXL2 overexpression and TGF-*β*1 treatment in 16HBE cells. **G** Relative protein levels of AKT and p-AKT compare to GAPDH after LOXL2 overexpression and TGF-*β*1 treatment in 16HBE cells. Data are shown as the means±SD of three independent experiments. ^*^P < 0.05, ^**^P < 0.01 versus the corresponding group

**Fig. 8:**
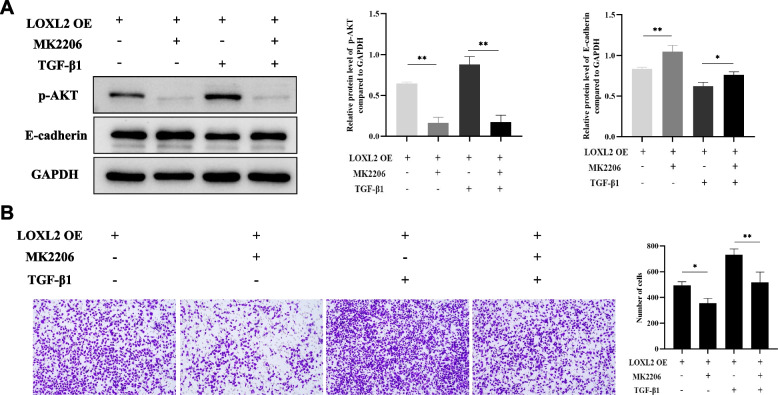
The roles of LOXL2 in airway epithelium may be mediated partly via the AKT signaling pathway. **A** Protein levels of p-AKT and E-cadherin in 16HBE cells treated with dimethyl sulfoxide, 5 μm MK2206, TGF-*β*1 plus dimethyl sulfoxide, or TGF-*β*1 plus MK2206 after transfection of LOXL2 overexpression plasmid. **B** Migration ability of 16HBE cells treated with dimethyl sulfoxide, 5 μm MK2206, TGF-*β*1 plus dimethyl sulfoxide, or TGF-*β*1 plus MK2206 after transfection of LOXL2 overexpression plasmid. Data are shown as the means ± SD of three independent experiments. ^*^P < 0.05, ^**^P < 0.01 versus the corresponding group

To clarify the underlying mechanisms of LOXL2 on airway remodeling, the KEGG pathway enrichment analysis of DEGs between OVA+LV2-shNC and OVA+LV2-shLOXL2 groups were performed (Fig. 7A-B). Apart from the enrichment items of focal adhesion and ECM-receptor interaction, we noticed that PI3K-AKT signaling pathway was significantly enriched (Fig. 7A). Meanwhile, based on the number of DEGs in the third circle of circular map, the mmu04151 contained the largest count of down-regulated DEGs, and the corresponding term of which was PI3K-AKT signaling pathway (Fig. 7B). Consistently, the knockdown of LOXL2 significantly inhibited the OVA-induced protein level of p-AKT in lung tissue (Fig. 7C). Further validation in 16HBE cells showed that LOXL2 knockdown could down-regulated the expression of AKT and p-AKT (Fig. 7D-E). Meanwhile, TGF-β1-induced upregulation of AKT and p-AKT was also significantly inhibited by LOXL2 silencing (Fig. 7D-E). Conversely, overexpression LOXL2 increased the protein levels of AKT and p-AKT, and also significantly enhanced the expression of TGF-β1-induced AKT and p-AKT (Fig. 7F-G). To further clarify the role of AKT signaling pathway in the development of airway remodeling associated with LOXL2, we treated 16HBE cells with LOXL2 overexpression plasmid, TGF-β1 and MK2206 (AKT inhibitor). Obviously, the protein level of p-AKT was reduced in the presence of MK2206 (Fig. 8A). Notably, the reduction of E-cadherin caused by LOXL2 overexpression was largely reversed with the inhibition of p-AKT (Fig. 8A). Meanwhile, the promoting impact of LOXL2 overexpression on migration ability of 16HBE cells also was abrogated under the treatment of MK2206 (Fig. 8B). Overall, the above findings support that the roles of LOXL2 in airway epithelium is mediated partly via AKT signaling pathway.

## Discussion

In this article, we noticed that LOXL2 is highly expressed in asthmatics (serum and airway epithelium) and OVA-induced asthma models (airway epithelium and lung tissues). Lentivirus-mediated knockdown of LOXL2 in vivo significantly inhibited OVA-induced ECM accumulation and EMT in mice. Moreover, the results of transfection experiments on 16HBE cells demonstrated that the trend of LOXL2 changes (overexpression or silencing) was consistent with that of remodeling and mesenchymal indicators. And the silencing of LOXL2 also reversed TGF-β1-induced remodeling and EMT process. Combining the RNA sequencing of mouse lung tissues and experiments in vitro, the above roles of LOXL2 in airway epithelium may be mediated partly via AKT signaling pathway.

At present, asthma still has a high incidence and economic burden, and its heterogeneity and repeatability determine that a considerable population will develop severe asthma, which is often accompanied by airway remodeling [5]. As an important airway structural cell, airway epithelium is the key participant in asthmatic airway remodeling. Briefly, apart from releasing a plethora of mediators involved in the initiation of remodeling, damaged airway epithelium also undergoes EMT during abnormal repair process [23]. Correspondingly, we analyzed the transcriptome changes of airway epithelial cells under pathological conditions in vitro and in vivo, and observed that LOXL2 gene was significantly upregulated in the airway epithelium cells of asthma. Subsequent experimental results confirmed the findings of bioinformatics analyses. Given that LOXL2 can be released extracellular, we detected serum LOXL2 level and found that it was significantly elevated in patients with asthma [24, 25]. Combined with the findings in biopsies from asthma patients, we speculate that airway epithelial cells may be an important source of serum LOXL2. Besides, we also found that the serum LOXL2 was negatively correlated with FEV1/FVC and FEV1%predicted. It follows that LOXL2 may play a role in the development of asthmatic airway remodeling.

In LOXL2, the highly conserved C-terminal consisting of a copper binding domain and a lysine-tyrosine-quinone cofactor domain supports the function of amine oxidase, that is, LOXL2 can catalyze oxidative deamination of primary amines to active aldehydes, thereby establishing covalent cross-linking between ECM proteins [10, 26]. On the other hand, the four scavenger receptor cysteine-rich (SRCR) domains at the N-terminal may allow LOXL2 to perform additional functions through ligand recognition and protein-protein interactions [26]. For example, Umana-Diaz et al. indicated that, independent of the catalytic activity, LOXL2 could directly bind with fibronectin and collagen IV to mediate ECM deposition and angiogenesis [27]. Previous studies have demonstrated that LOXL2 contributed to pathologic fibrosis in a variety of diseases [11, 16–18]. Consistently, our study highlighted the roles of LOXL2 in airway remodeling, namely, LOXL2 knockdown significantly reduced ECM accumulation (collagen I and α-SMA) in OVA-induced mouse models.

Meanwhile, the results of transcriptome sequencing and transfection experiments suggested LOXL2 regulated cell adhesion and migration, which may partially attribute to its interactions with various proteins, such as vimentin, ezrin, fascin, and tropomodulin [28, 29]. Given that the changes of cell adhesion and motility ability are important features of EMT process, we found that LOXL2 intervention in vivo did affect the expression of EMT indicators in lung tissues of OVA-treated mice. In vitro, after LOXL2 overexpression in bronchial epithelial cells, the level of E-cadherin was decreased, while the level of N-cadherin and fibronectin was increased. In return, the LOXL2 knockdown increased E-cadherin expression. These are consistent with prior studies, specifically, LOXL2 induced EMT in multiple cancer cell lines through protecting snail1 from phosphorylation, ubiquitination, and subsequent degradation [30]. Moreover, the remodeled ECM mediated by enzyme activity of LOXL2 also mechanically drived the emergence of mesenchymal phenotypes [31, 32].

Notably, TGF-β1 is a key driver of airway remodeling in asthma [33]. Existing studies found that after treatment with TGF-β, multiple cells including smooth muscle cells and fibroblasts are activated to highly express LOXL2 [20, 34, 35]. Meanwhile, the LOXL2 knockdown reduced the expression of TGF-β target genes (COL1A1, FN1 and α-SMA) in fibroblasts [11]. Based on these, we used TGF-β1 to induced the airway remodeling and EMT cellular model, and found that the expression of LOXL2 was significantly up-regulated. Furthermore, the LOXL2 silencing in bronchial epithelial cells weakened the TGF-β1-induced EMT and remodeling, while it could be enhanced after LOXL2 overexpression. The results suggested that as an effector molecule of TGF-β1, LOXL 2 is a powerful booster of EMT and remodeling. Sethi et al. have proposed that both canonical Smad and non-Smad JNK/AP-1 signaling pathways medicated the TGF-β1 induction of LOXL2 gene in trabecular meshwork cells [36]. In the future, it is necessary to perform relevant experiments to determine whether the above relationship is also observed in bronchial epithelial cells.

It has been reported that LOXL2 regulates endothelium-interstitial transformation during angiogenesis through the protein kinase B (PKB)/Akt and focal adhesion kinase (FAK) signaling [37]. Recently, Fan et al. found that LOXL2 promotes the proliferation, invasion, and metastasis of esophageal squamous carcinoma by increasing p-AKT expression [38]. Researchers also learned that the activation of AKT is associated with the pathogenesis of several respiratory diseases, including asthma [39–41]. In our study, the KEGG pathway enrichment analysis of RNA-sequencing indicated that LOXL2 knockdown in OVA-induced asthma models significantly affected the PI3K-AKT signaling pathway, which contains the most down-regulated DEGs. Subsequent experiments proved that the alteration of LOXL2 in 16HBE cells significantly affected the expression and activation of AKT, concretely, LOXL2 knockdown decreased the levels of AKT and p-AKT, while LOXL2 overexpression increased the levels of AKT and p-AKT. Furthermore, the inhibition of AKT partially alleviated the consequences associated with LOXL2 overexpression.

Admittedly, certain limitations exist in our study. First, the relationship between the role of LOXL2 in airway epithelium and its enzyme activity has not been explored in detail. Additionally, in light of bidirectional interaction between inflammation and remodeling of airway epithelium in asthma, future studies should also explorer the effects and mechanisms of LOXL2 on airway inflammation. Finally, several novel functions of LOXL2, such as regulating transcription and epigenetic inheritance, have recently been identified, which is worthy of further investigation in asthma.

## Conclusions

Taken together, we demonstrated that LOXL2 is increased in the airway epithelium of asthma, and regulates EMT and ECM deposition partly via the AKT signaling pathway. These results provide further evidence that LOXL2 is involved in airway remodeling and deserves to be considered as a potential therapeutic target for asthma.

### Supplementary Information

Additional file 1: Table. S1. List of extracellular matrix genes. Table. S2. The clinical characteristics of subjects that donated the bronchial epithelial specimens. Table. S3. Primers for qRT-PCR. Table. S4. Characteristics of subjects in serum ELISA study. Fig. S1. Identification and confirmation of DEGs. (A, B) Volcano plots of DEGs in two datasets (GSE179156 and GSE40374). Up-regulated DEGs are highlighted in red, and down-regulated DEGs are highlighted in blue. (C) Venn diagram indicating the intersection of ECM-related genes and DEGs in the above two datasets. (D)The interaction network of 44 overlapping genes based on STING database. (E) The Identification of key genes using five algorithms (MCC, MNC, EPC, DMNC and Degree) in Cytoscape software. (F) LOXL2 protein expression levels of normal tissues from Human Protein Atlas database. (G) Heat map of LOXL2 differentially expressed across different cell types determined by the Human Protein Atlas database. (H, I) Differential expression analysis of LOXL2 in GSE179156 and GSE40374 (Student’s t test or Mann-Whitney test were used). *P < 0.05, **P < 0.01 versus the control group. Fig. S2. Validation of LOXL2 knockdown in vivo. (A) Lentivirus-packed shLOXL2 significantly reduced the protein level of LOXL2 in mouse lung tissue. (B, C) The IF staining of lung tissue sections suggested that after treatment with LV2-shLOXL2, the LOXL2 in airway epithelium of OVA-induced models was inhibited. Scale bar, 50*µ*m. Data are shown as the means±SD of three independent experiments. *P < 0.05, **P < 0.01 versus the corresponding group.

## Data Availability

All data used or analyzed in this study are available from the corresponding author.

## Abbreviations

ECM: extracellular matrix
LOXL2: Lysyl oxidase like 2
ASM: airway smooth muscle
GEO: Gene Expression Omnibus
TGF-*β*1: transforming growth factor-*β*1
ELISA: Enzyme-linked immunosorbent assay
KM: Keratinocyte Medium
KGS: Keratinocyte Growth Supplement
siRNA: small interfering RNA
LOXL2 OE: LOXL2 overexpression
OVA: ovalbumin
shRNA: short hairpin RNA
DEGs: differentially expressed genes
GO: Gene Ontology
KEGG: Kyoto Encyclopedia of Genes and Genomes
qRT-PCR: Quantitative real-time-polymerase chain reaction
*α*-SMA: alpha smooth muscle actin
IHC: Immunohistochemistry
IF: Immunofluorescence
FEV1: forced expiratory volume in one second
FVC: forced vital capacity ratio
EMT: epithelial-mesenchymal transition
PI3K: Phosphatidylinositol-3-kinase
PKB: protein kinase B
AKT: serine-threonine protein kinase
p-AKT: phospho-AKT

## References

[1] Mortimer K, Lesosky M, García-Marcos L, Asher MI, Pearce N, Ellwood E (2022). The burden of asthma, hay fever and eczema in adults in 17 countries: GAN Phase I study. Eur Respir J..

[2] Asher MI, Rutter CE, Bissell K, Chiang CY, El Sony A, Ellwood E (2021). Worldwide trends in the burden of asthma symptoms in school-aged children: Global Asthma Network Phase I cross-sectional study. Lancet..

[3] Mims JW. Asthma: definitions and pathophysiology. Int Forum Allergy Rhinol. 2015;5(S1). 10.1002/alr.21609. 10.1002/alr.21609 26335832

[4] Global Initiative for Asthma (GINA). Global Strategy for Asthma Management and Prevention. 2023. Updated July 2023. https://ginasthma.org/2023-gina-main-report/.

[5] Varricchi G, Ferri S, Pepys J, Poto R, Spadaro G, Nappi E (2022). Biologics and airway remodeling in severe asthma. Allergy..

[6] Burgstaller G, Oehrle B, Gerckens M, White ES, Schiller HB, Eickelberg O (2017). The instructive extracellular matrix of the lung: basic composition and alterations in chronic lung disease. Eur Respir J..

[7] Karakioulaki M, Papakonstantinou E, Stolz D (2020). Extracellular matrix remodelling in COPD. Eur Respir Rev..

[8] Guo T, He C, Venado A, Zhou Y. Extracellular Matrix Stiffness in Lung Health and Disease. Wiley; 2022. 10.1002/cphy.c210032. 10.1002/cphy.c210032 PMC10088466 35766837

[9] Parker MW, Rossi D, Peterson M, Smith K, Sikström K, White ES (2014). Fibrotic extracellular matrix activates a profibrotic positive feedback loop. J Clin Investig..

[10] Moon HJ, Finney J, Ronnebaum T, Mure M (2014). Human lysyl oxidase-like 2. Bioorg Chem..

[11] Yang J, Savvatis K, Kang JS, Fan P, Zhong H, Schwartz K, et al. Targeting LOXL2 for cardiac interstitial fibrosis and heart failure treatment. Nat Commun. 2016;7(1). 10.1038/ncomms13710. 10.1038/ncomms13710 PMC5171850 27966531

[12] Erasmus M, Samodien E, Lecour S, Cour M, Lorenzo O, Dludla P (2020). Linking LOXL2 to Cardiac Interstitial Fibrosis. Int J Mol Sci..

[13] Schietke R, Warnecke C, Wacker I, Schödel J, Mole DR, Campean V (2010). The Lysyl Oxidases LOX and LOXL2 Are Necessary and Sufficient to Repress E-cadherin in Hypoxia. J Biol Chem..

[14] Wen B, Xu LY, Li EM (2020). LOXL2 in cancer: regulation, downstream effectors and novel roles. Biochim Biophys Acta (BBA) Rev Cancer.

[15] Nave AH, Mižíková I, Niess G, Steenbock H, Reichenberger F, Talavera ML (2014). Lysyl Oxidases Play a Causal Role in Vascular Remodeling in Clinical and Experimental Pulmonary Arterial Hypertension. Arterioscler Thromb Vasc Biol..

[16] Ikenaga N, Peng ZW, Vaid KA, Liu SB, Yoshida S, Sverdlov DY (2017). Selective targeting of lysyl oxidase-like 2 (LOXL2) suppresses hepatic fibrosis progression and accelerates its reversal. Gut..

[17] Wen X, Liu Y, Bai Y, Li M, Fu Q, Zheng Y. LOXL2, a copper-dependent monoamine oxidase, activates lung fibroblasts through the TGF-$$\beta$$,/Smad pathway. Int J Mol Med. 2018. 10.3892/ijmm.2018.3927. 10.3892/ijmm.2018.3927 30320382

[18] Cosgrove D, Dufek B, Meehan DT, Delimont D, Hartnett M, Samuelson G, et al. Lysyl oxidase like-2 contributes to renal fibrosis in Col4$$\alpha$$3/Alport mice. Kidney Int. 2018;94(2):303–14. 10.1016/j.kint.2018.02.024. 10.1016/j.kint.2018.02.024 PMC7523185 29759420

[19] Wu Y, Wu Y, Yang Y, Yu J, Wu J, Liao Z, et al. Lysyl oxidase-like 2 inhibitor rescues D-galactose-induced skeletal muscle fibrosis. Aging Cell. 2022;21(7). 10.1111/acel.13659. 10.1111/acel.13659 PMC9282848 35712918

[20] Ramis J, Middlewick R, Pappalardo F, Cairns JT, Stewart ID, John AE (2022). Lysyl oxidase like 2 is increased in asthma and contributes to asthmatic airway remodelling. Eur Respir J..

[21] Burgess JK, Weiss DJ, Westergren-Thorsson G, Wigen J, Dean CH, Mumby S (2024). Extracellular Matrix as a Driver of Chronic Lung Diseases. Am J Respir Cell Mol Biol..

[22] Andersson-Sjoland A, Hallgren O, Rolandsson S, Weitoft M, Tykesson E, Larsson-Callerfelt AK (2014). Versican in inflammation and tissue remodeling: The impact on lung disorders. Glycobiology..

[23] Chatziparasidis G, Bush A, Chatziparasidi MR, Kantar A (2023). Airway epithelial development and function: A key player in asthma pathogenesis?. Paediatr Respir Rev..

[24] Chien JW, Richards TJ, Gibson KF, Zhang Y, Lindell KO, Shao L (2013). Serum lysyl oxidase-like 2 levels and idiopathic pulmonary fibrosis disease progression. Eur Respir J..

[25] Zhao Y, Tang K, Tianbao X, Wang J, Yang J, Li D. Increased serum lysyl oxidase-like 2 levels correlate with the degree of left atrial fibrosis in patients with atrial fibrillation. Biosci Rep. 2017;37(6). 10.1042/bsr20171332. 10.1042/BSR20171332 PMC5696452 29089463

[26] Molnar J, Fong KSK, He QP, Hayashi K, Kim Y, Fong SFT (2003). Structural and functional diversity of lysyl oxidase and the LOX-like proteins. Biochim Biophys Acta (BBA) Protein Proteomics.

[27] Bignon M, Pichol-Thievend C, Hardouin J, Malbouyres M, Bréchot N, Nasciutti L (2011). Lysyl oxidase-like protein-2 regulates sprouting angiogenesis and type IV collagen assembly in the endothelial basement membrane. Blood..

[28] Almacellas-Rabaiget O, Monaco P, Huertas-Martinez J, García-Monclús S, Chicón-Bosch M, Maqueda-Marcos S (2020). LOXL2 promotes oncogenic progression in alveolar rhabdomyosarcoma independently of its catalytic activity. Cancer Lett..

[29] Zhan XH, Jiao JW, Zhang HF, Xu XE, He JZ, Li RL (2019). LOXL2 Upregulates Phosphorylation of Ezrin to Promote Cytoskeletal Reorganization and Tumor Cell Invasion. Cancer Res..

[30] Moon HJ, Finney J, Xu L, Moore D, Welch DR, Mure M (2013). MCF-7 Cells Expressing Nuclear Associated Lysyl Oxidase-like 2 (LOXL2) Exhibit an Epithelial-to-Mesenchymal Transition (EMT) Phenotype and Are Highly Invasive in Vitro. J Biol Chem..

[31] Rice AJ, Cortes E, Lachowski D, Cheung BCH, Karim SA, Morton JP (2017). Matrix stiffness induces epithelial-mesenchymal transition and promotes chemoresistance in pancreatic cancer cells. Oncogenesis..

[32] Brown AC, Fiore VF, Sulchek TA, Barker TH (2012). Physical and chemical microenvironmental cues orthogonally control the degree and duration of fibrosis-associated epithelial-to-mesenchymal transitions. J Pathol..

[33] Halwani R, Al-Muhsen S, Al-Jahdali H, Hamid Q. Role of Transforming Growth Factor-$$\beta$$, in Airway Remodeling in Asthma. Am J Respir Cell Mol Biol. 2011;44(2):127–33. 10.1165/rcmb.2010-0027tr. 10.1165/rcmb.2010-0027TR 20525803

[34] Aumiller V, Strobel B, Romeike M, Schuler M, Stierstorfer BE, Kreuz S. Comparative analysis of lysyl oxidase (like) family members in pulmonary fibrosis. Sci Rep. 2017;7(1). 10.1038/s41598-017-00270-0. 10.1038/s41598-017-00270-0 PMC5428068 28273952

[35] Semkova ME, Hsuan JJ. TGF$$\beta$$,-1 Induced Cross-Linking of the Extracellular Matrix of Primary Human Dermal Fibroblasts. Int J Mol Sci. 2021;22(3):984. 10.3390/ijms22030984. 10.3390/ijms22030984 PMC7863744 33498156

[36] Sethi A, Mao W, Wordinger RJ, Clark AF. Transforming Growth Factor-$$\beta$$, Induces Extracellular Matrix Protein Cross-Linking Lysyl Oxidase (LOX) Genes in Human Trabecular Meshwork Cells. Invest Opthalmology Vis Sci. 2011;52(8):5240. 10.1167/iovs.11-7287. 10.1167/iovs.11-7287 PMC3176072 21546528

[37] de Jong OG, van der Waals LM, Kools FRW, Verhaar MC, van Balkom BWM (2018). Lysyl oxidase-like 2 is a regulator of angiogenesis through modulation of endothelial-to-mesenchymal transition. J Cell Physiol..

[38] Fan Z, Liu Y, Liu X, Nian W, Huang X, Yang Q (2023). Phosphorylation of AKT by lysyl oxidase-like 2 activates the PI3K/AKT signaling pathway to promote proliferation, invasion and metastasis in esophageal squamous carcinoma. Clin Transl Oncol..

[39] Liu Y, Kong H, Cai H, Chen G, Chen H, Ruan W. Progression of the PI3K/Akt signaling pathway in chronic obstructive pulmonary disease. Front Pharmacol. 2023;14. 10.3389/fphar.2023.1238782. 10.3389/fphar.2023.1238782 PMC10548138 37799975

[40] Wang J, Hu K, Cai X, Yang B, He Q, Wang J (2022). Targeting PI3K/AKT signaling for treatment of idiopathic pulmonary fibrosis. Acta Pharm Sin B..

[41] Du L, Xu C, Tang K, Shi J, Tang L, Lisha X (2023). Epithelial CST1 Promotes Airway Eosinophilic Inflammation in Asthma via the AKT Signaling Pathway. Allergy Asthma Immunol Res..

