# Supplementary figures and images for: Targeting lysyl oxidase like 2 attenuates OVA-induced airway remodeling partly via the AKT signaling pathway
Source: Respir Res. 2024 Jun 1;25:230. doi: 10.1186/s12931-024-02811-4 (PMC11144323; doi:10.1186/s12931-024-02811-4)

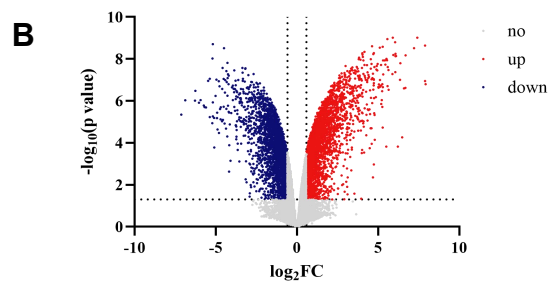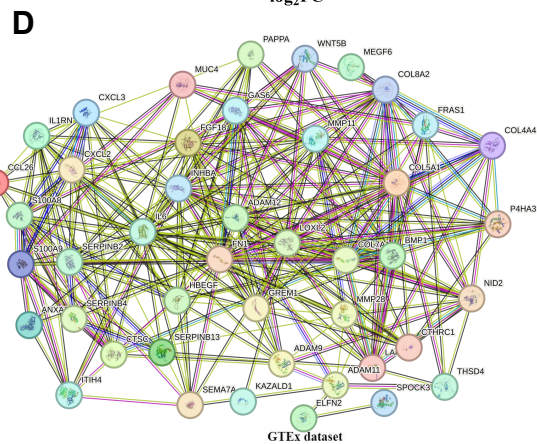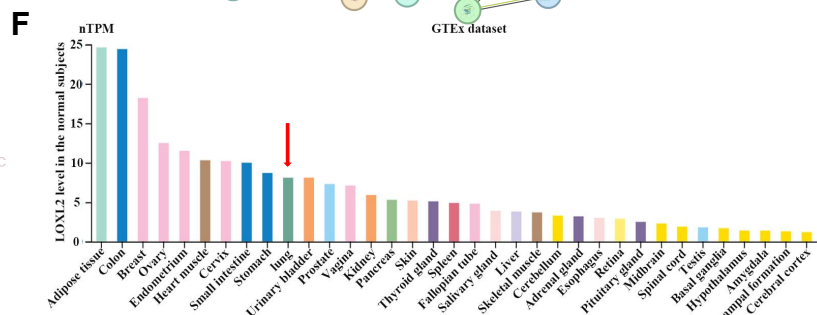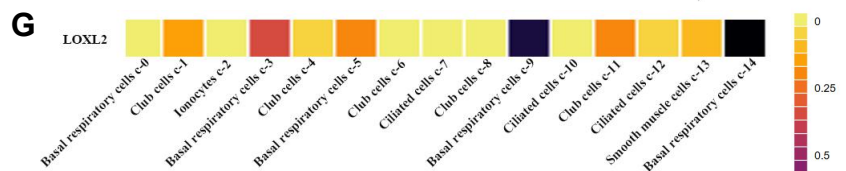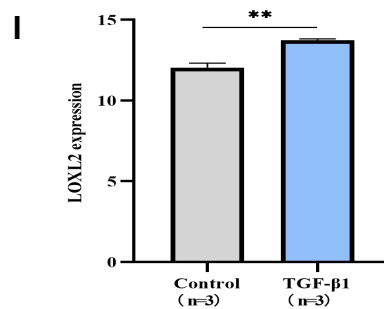

Supplement: Supplementary file 1 — Additional file 1: Table. S1. List of extracellular matrix genes. Table. S2. The clinical characteristics of subjects that donated the bronchial epithelial specimens. Table. S3. Primers for qRT-PCR. Table. S4. Characteristics of subjects in serum ELISA study. Fig. S1. Identification and confirmation of DEGs. (A, B) Volcano plots of DEGs in two datasets (GSE179156 and GSE40374). Up-regulated DEGs are highlighted in red, and down-regulated DEGs are highlighted in blue. (C) Venn diagram indicating the intersection of ECM-related genes and DEGs in the above two datasets. (D)The interaction network of 44 overlapping genes based on STING database. (E) The Identification of key genes using five algorithms (MCC, MNC, EPC, DMNC and Degree) in Cytoscape software. (F) LOXL2 protein expression levels of normal tissues from Human Protein Atlas database. (G) Heat map of LOXL2 differentially expressed across different cell types determined by the Human Protein Atlas database. (H, I) Differential expression analysis of LOXL2 in GSE179156 and GSE40374 (Student’s t test or Mann-Whitney test were used). *P < 0.05, **P < 0.01 versus the control group. Fig. S2. Validation of LOXL2 knockdown in vivo. (A) Lentivirus-packed shLOXL2 significantly reduced the protein level of LOXL2 in mouse lung tissue. (B, C) The IF staining of lung tissue sections suggested that after treatment with LV2-shLOXL2, the LOXL2 in airway epithelium of OVA-induced models was inhibited. Scale bar, 50µm. Data are shown as the means±SD of three independent experiments. *P < 0.05, **P < 0.01 versus the corresponding group. [file 12931_2024_2811_MOESM1_ESM.zip › Additional file 1 Fig S1.pdf]

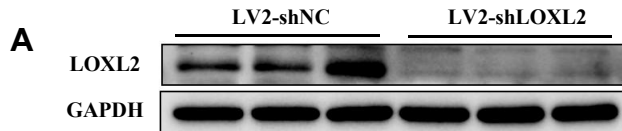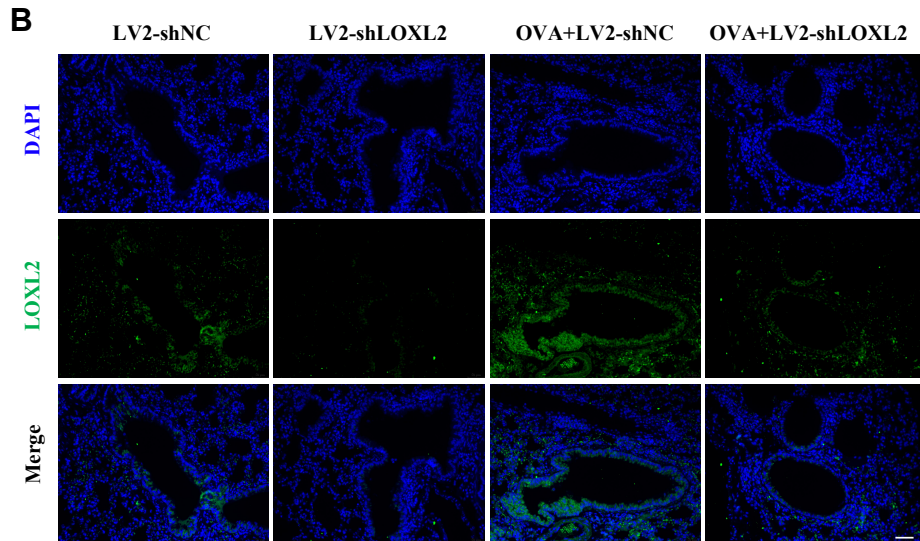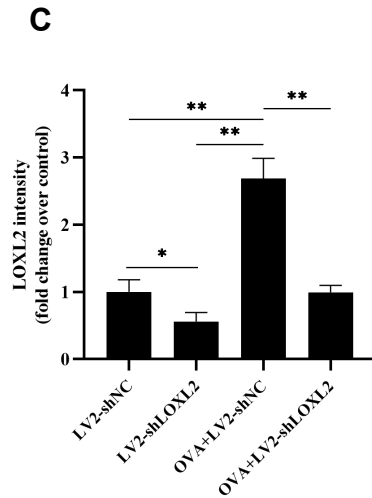

Supplement: Supplementary file 1 — Additional file 1: Table. S1. List of extracellular matrix genes. Table. S2. The clinical characteristics of subjects that donated the bronchial epithelial specimens. Table. S3. Primers for qRT-PCR. Table. S4. Characteristics of subjects in serum ELISA study. Fig. S1. Identification and confirmation of DEGs. (A, B) Volcano plots of DEGs in two datasets (GSE179156 and GSE40374). Up-regulated DEGs are highlighted in red, and down-regulated DEGs are highlighted in blue. (C) Venn diagram indicating the intersection of ECM-related genes and DEGs in the above two datasets. (D)The interaction network of 44 overlapping genes based on STING database. (E) The Identification of key genes using five algorithms (MCC, MNC, EPC, DMNC and Degree) in Cytoscape software. (F) LOXL2 protein expression levels of normal tissues from Human Protein Atlas database. (G) Heat map of LOXL2 differentially expressed across different cell types determined by the Human Protein Atlas database. (H, I) Differential expression analysis of LOXL2 in GSE179156 and GSE40374 (Student’s t test or Mann-Whitney test were used). *P < 0.05, **P < 0.01 versus the control group. Fig. S2. Validation of LOXL2 knockdown in vivo. (A) Lentivirus-packed shLOXL2 significantly reduced the protein level of LOXL2 in mouse lung tissue. (B, C) The IF staining of lung tissue sections suggested that after treatment with LV2-shLOXL2, the LOXL2 in airway epithelium of OVA-induced models was inhibited. Scale bar, 50µm. Data are shown as the means±SD of three independent experiments. *P < 0.05, **P < 0.01 versus the corresponding group. [file 12931_2024_2811_MOESM1_ESM.zip › Additional file 1 Fig S2.pdf]
